# Correlating the triglyceride glucose index with short-term neurological and functional prognosis following intravenous thrombolysis in acute ischemic stroke patients

**DOI:** 10.3389/fneur.2025.1670811

**Published:** 2025-10-07

**Authors:** Defeng Hua, Zhen Guo

**Affiliations:** ^1^Department of Neurology, Weifang People’s Hospital, Weifang, China; ^2^Department of Clinical Laboratory, Weifang People’s Hospital, Weifang, China

**Keywords:** triglyceride glucose index, acute ischemic stroke, intravenous thrombolysis, neurological outcomes, functional outcomes

## Abstract

**Objective:**

To assess the correlation between the triglyceride glucose (TyG) index and short-term neurological and functional outcomes in patients with acute ischemic stroke (AIS) post-intravenous thrombolysis (IVT).

**Methods:**

This prospective observational study included AIS patients treated with IVT within 4.5 h from symptom onset. The TyG index was calculated using fasting triglyceride and glucose levels. Neurological improvement was evaluated by a reduction in National Institutes of Health Stroke Scale (NIHSS) scores, and functional outcome by modified Rankin Scale (mRS) at discharge. Statistical analysis included correlation and regression analyses.

**Results:**

Among 150 AIS patients, the TyG index significantly correlated with both NIHSS (rho = 0.45, *p* < 0.01) and mRS (rho = 0.38, *p* < 0.01) scores at discharge. A higher TyG index was associated with neurological non-improvement (OR = 2.11, *p* = 0.002) and poor functional outcomes (OR = 1.89, *p* = 0.005) after adjustment for confounders.

**Conclusion:**

The TyG index is significantly associated with short-term outcomes in AIS patients post-IVT, suggesting its potential as a prognostic marker for stroke severity and recovery. Future studies with larger cohorts are needed to confirm these findings.

## 1 Introduction

Stroke remains a leading cause of long-term disability and mortality globally, with acute ischemic stroke (AIS) accounting for approximately 80% of all stroke cases (1). The administration of intravenous thrombolysis (IVT) within 4.5 h of symptom onset is a standard treatment for AIS, aimed at recanalizing the occluded vessel and salvaging ischemic penumbra (2, 3). Metabolic health has emerged as a critical factor in stroke prognosis, with insulin resistance and dyslipidemia implicated in the pathogenesis of AIS (4). The triglyceride glucose (TyG) index, a novel and easily obtainable biomarker derived from fasting triglyceride and glucose levels, has been proposed as a reliable indicator of insulin resistance and metabolic dysregulation (5). Recent studies have demonstrated the TyG index’s association with an increased risk of cardiovascular events and poor outcomes in various clinical settings, including stroke (6, 7).

The TyG index’s potential role in predicting outcomes in AIS patients post-IVT is an area of growing interest. Elevated TyG index values have been linked to a higher risk of stroke incidence and mortality, suggesting that the TyG index may serve as a valuable predictor of short-term neurological and functional prognosis following IVT (8, 9). Understanding the relationship between the TyG index and stroke outcomes could enhance our predictive capabilities, inform treatment decisions, and potentially improve functional recovery and reduce disability post-stroke.

This study aims to investigate the correlation between the TyG index and short-term neurological and functional prognosis in AIS patients following IVT. By elucidating the role of the TyG index in stroke outcomes, this research contributes to the growing body of evidence on the importance of metabolic biomarkers in stroke management and prognosis.

## 2 Methods

### 2.1 Study design and population

The prospective observational study included patients diagnosed with acute ischemic stroke (AIS) who underwent intravenous thrombolysis (IVT) at our tertiary care center from January 2022–January 2024. Eligible participants were adults aged 18 years or older with a confirmed diagnosis of AIS, treated with IVT within 4.5 h of symptom onset, and had a baseline National Institutes of Health Stroke Scale (NIHSS) score of 4 or higher, indicating moderate to severe stroke severity.

The sample size was determined based on the availability of eligible patients during the study period (January 2022–January 2024) and the feasibility of data collection. A *post-hoc* power analysis was conducted using the observed effect size (OR = 2.11), which indicated that the study had >80% power to detect a significant association between TyG index and neurological outcomes at *α* = 0.05.

### 2.2 Data collection

We collected demographic data, medical history, and laboratory parameters, including fasting glucose and triglyceride levels, at admission. The triglyceride glucose (TyG) index was calculated using the formula (10):


TYG=ln[fasting triglycerides(mg/dL)×fasting glucose(mg/dL)/2]

Baseline NIHSS scores and modified Rankin Scale (mRS) at discharge were recorded to assess neurological and functional outcomes, respectively. Stroke mechanism was determined based on Trial of ORG 10172 in Acute Stroke Treatment (TOAST) classification. Infarct volume was measured using brain imaging (CT or MRI) at admission.

### 2.3 Neurological and functional outcomes assessment

The NIHSS is a 15-item neurological examination scale used to quantify stroke severity, with scores ranging from 0 to 42; higher scores indicate more severe neurological deficits. Neurological improvement was defined as a reduction of at least 18% in NIHSS scores from admission to discharge, indicating improve of neurological function (11). The mRS is a 6-point disability scale ranging from 0 (no symptoms) to 6 (death), commonly used to assess functional recovery after stroke. A favorable functional outcome was defined as an mRS score of 0–2 at discharge, indicating the ability to be independent in daily activities. Patients were categorized into neurological improved (NI) and neurological unimproved (NU) groups, as well as favorable functional outcome (FFO) and poor functional outcome (PFO) groups based on predefined criteria.

### 2.4 Statistical analysis

The study compared the TyG index in patients with favorable and unfavorable outcomes using Student’s *t*-test or Mann–Whitney *U*-test for continuous variables, and chi-square test for categorical variables. The correlation between the TyG index and outcomes was assessed using Spearman’s rank correlation coefficient. Multiple logistic regression analysis was performed to adjust for potential confounders including age, hypertension, and diabetes, current smoking, prior stroke, atrial fibrillation, total cholesterol, triglycerides, HDL cholesterol, LDL cholesterol, homocysteine (HCY) and C-reactive protein (CRP). Subgroup analyses were conducted to investigate the relationship between the TyG index and short-term neurological and functional outcomes in specific subsets.

The statistical analysis was performed using R version 4.2.3. A *p*-value less than 0.05 was considered to indicate statistical significance.

## 3 Results

### 3.1 Study population characteristics

The baseline characteristics of 150 AIS patients post-IVT, stratified by neurological improvement and functional outcome status were presented in Tables 1, 2.

**Table 1:** Baseline characteristics by neurological outcome group.

Characteristic	Neurological improved group (NI group, *N* = 97)	Neurological unimproved group (NU group, *N* = 53)	*p*-value
Age, years	61.5 ± 10.8	63.2 ± 10.2	0.31
Male, *n* (%)	56 (58)	31 (58)	0.96
Hypertension, *n* (%)	67 (69)	38 (72)	0.58
Diabetes, *n* (%)	28 (29)	17 (32)	0.53
Current smoker, *n* (%)	22 (23)	16 (30)	0.34
Prior stroke, *n* (%)	14 (14)	11 (21)	0.15
Atrial fibrillation, *n* (%)	21 (22)	18 (34)	0.07
Total cholesterol, mg/dL	192.5 ± 35.7	201.3 ± 42.1	0.08
Triglycerides, mg/dL	123.4 (67.2–183.6)	138.5 (76.4–206.8)	0.06
HDL cholesterol, mg/dL	47.6 ± 15.3	44.2 ± 14.8	0.21
LDL cholesterol, mg/dL	116.3 ± 30.5	124.6 ± 35.2	0.11
Homocysteine (HCY), μmol/L	12.4 (4.7–20.1)	14.1 (5.2–22.3)	0.02
CRP, mg/L	3.5 (2.8–5.1)	4.2 (3.1–6.3)	0.04
NIHSS score, median (IQR)	7 (5–10)	10 (8–13)	<0.01
TyG index, median (IQR)	8.2 (7.6–8.9)	9.1 (8.4–9.9)	<0.01
TOAST classification, *n* (%)			
Large artery atherosclerosis	21 (22)	16 (30)	0.24
Cardioembolism	34 (35)	19 (36)	0.87
Small vessel occlusion	24 (25)	11 (21)	0.55
Other determined etiology	7 (7)	3 (6)	0.78
Undetermined etiology	11 (11)	4 (8)	0.65
Stroke mechanism, *n* (%)			
Large vessel occlusion	30 (31)	20 (38)	0.12
Small vessel disease	20 (21)	10 (19)	0.34
Infarct volume, median (IQR)	15 (10–20)	25 (20–35)	<0.01

**Table 2:** Baseline characteristics by functional outcome group.

Characteristic	Favorable functional outcome group (FFO group, *N* = 70)	Poor functional outcome group (PFO group, *N* = 80)	*p*-value
Age, years	60.9 ± 11.1	63.6 ± 10.3	0.09
Male, *n* (%)	41 (59)	46 (58)	0.89
Hypertension, *n* (%)	49 (70)	56 (70)	0.99
Diabetes, *n* (%)	21 (30)	24 (30)	0.97
Current smoker, *n* (%)	15 (21)	24 (30)	0.19
Prior stroke, *n* (%)	9 (13)	18 (23)	0.08
Atrial fibrillation, *n* (%)	13 (19)	28 (35)	0.02
Total cholesterol, mg/dL	195.6 ± 37.1	198.2 ± 40.5	0.68
Triglycerides, mg/dL	117.3 (62.4–172.5)	136.7 (79.1–216.4)	0.07
HDL cholesterol, mg/dL	49.8 ± 16.2	45.5 ± 14.5	0.14
LDL cholesterol, mg/dL	119.4 ± 32.3	122.1 ± 34.8	0.59
Homocysteine (HCY), μmol/L	11.9 (4.3–18.7)	13.7 (5.4–21.5)	0.01
CRP, mg/L	3.2 (2.5–4.9)	3.9 (2.9–5.9)	0.03
NIHSS score, median (IQR)	6 (4–9)	9 (7–12)	<0.01
TyG index, median (IQR)	8.3 (7.7–9.0)	8.9 (8.2–9.7)	0.02
TOAST classification, *n* (%)			
Large artery atherosclerosis	18 (26)	22 (28)	0.76
Cardioembolism	25 (36)	28 (35)	0.91
Small vessel occlusion	19 (27)	19 (24)	0.67
Other determined etiology	6 (9)	8 (10)	0.72
Undetermined etiology	8 (12)	13 (16)	0.39
Stroke mechanism, *n* (%)			
Large vessel occlusion	25 (36)	25 (31)	0.67
Small vessel disease	15 (21)	15 (19)	0.78
Infarct volume, median (IQR)	12 (8–18)	22 (18–30)	<0.01

The NI group (*N* = 97) and NU group (*N* = 53) did not show a statistically significant difference in age, gender, prevalence of hypertension, and diabetes (*p* > 0.05 for all). However, the NU group exhibited a higher proportion of patients with atrial fibrillation (34% vs. 22%, *p* = 0.07) and higher levels of homocysteine (HCY) (14.1 μmol/L vs. 12.4 μmol/L, *p* = 0.02) and CRP (4.2 mg/L vs. 3.5 mg/L, *p* = 0.04). NIHSS scores and TyG index were significantly higher in the NU group (*p* < 0.01 for both), indicating greater stroke severity and metabolic dysregulation in non-improved patients.

The FFO group (*N* = 70) and PFO group (*N* = 80) did not differ significantly in age, gender, hypertension, diabetes, and smoking status (*p* > 0.05 for all). Atrial fibrillation was more prevalent in the PFO group (35% vs. 19%, *p* = 0.02). Elevated HCY (13.7 μmol/L vs. 11.9 μmol/L, *p* = 0.01) and CRP levels (3.9 mg/L vs. 3.2 mg/L, *p* = 0.03) were also associated with poor functional outcomes. Consistent with the neurological outcome group, higher NIHSS scores and TyG index were observed in the PFO group (*p* < 0.01 for both).

TOAST classification distribution was similar across both the NI and NU groups, as well as the FFO and PFO groups, with no significant differences in stroke etiology (*p* > 0.05 for all comparisons).

### 3.2 Correlation analysis of TyG index with NIHSS and mRS scores at discharge

The TyG index demonstrated a significant positive correlation with the NIHSS score at discharge (Spearman’s rho = 0.45, *p* < 0.01), indicating that higher TyG index values were associated with greater neurological impairment. Similarly, the TyG index showed a significant positive correlation with the mRS score at discharge (Spearman’s rho = 0.38, *p* < 0.01), suggesting that higher TyG index values were linked to poorer functional outcomes.

### 3.3 Association of TyG index with neurological and functional outcomes

The TyG index was significantly associated with neurological unimproved status (OR = 2.11, 95% CI: 1.33–3.35, *p* = 0.002) after adjusting for the covariates. This indicates that for each unit increase in the TyG index, the odds of not experiencing neurological improvement were more than double. Similarly, the TyG index was significantly associated with poor functional outcome (OR = 1.89, 95% CI: 1.21–2.95, *p* = 0.005) after adjustment. This suggests that a higher TyG index is associated with an increased likelihood of poor functional recovery post-stroke (Table 3).

**Table 3:** Multiple logistic regression analysis for neurological and functional outcomes.

Variable	Neurological outcome OR (95% CI)	*p*-value	Functional outcome OR (95% CI)	*p*-value
TyG index	2.11 (1.33–3.35)	0.002	1.89 (1.21–2.95)	0.005
Age	1.04 (1.01–1.07)	0.01	1.05 (1.02–1.08)	0.01
Atrial fibrillation	2.47 (1.48–4.12)	<0.01	2.15 (1.29–3.58)	0.01
CRP	1.82 (1.11–2.98)	0.02	—	—
Hypertension	—	—	1.23 (0.76–2.00)	0.40
Diabetes	0.79 (0.49–1.27)	0.33	1.12 (0.68–1.85)	0.65
LDL cholesterol	1.02 (0.99–1.05)	0.21	0.98 (0.95–1.02)	0.36
HCY	1.15 (1.02–1.29)	0.03	1.20 (1.06–1.36)	0.01
Infarct volume	1.03 (1.01–1.05)	0.004	1.04 (1.02–1.06)	0.002

### 3.4 Association between TyG index and stroke outcomes across subgroups

The analysis revealed that among patients aged 65 and above, there was a pronounced and significant correlation between the TyG index and both neurological and functional outcomes, which was more pronounced than in their younger counterparts. Across genders, the TyG index showed a robust association with outcomes, with no discernible differences between male and female patients.

In terms of comorbidities, hypertension was found to be a factor that significantly influenced the correlation between the TyG index and stroke outcomes. Furthermore, the presence of diabetes amplified the association between the TyG index and outcomes, suggesting a heightened relevance of the TyG index in diabetic stroke patients.

Smoking habits also played a role, as current smokers displayed a significant association between the TyG index and outcomes, in contrast to never/former smokers who did not exhibit such a correlation.

In summary, the relationship between the TyG index and post-stroke outcomes is shaped by various patient characteristics, including age, presence of hypertension, diabetes, and smoking status (Table 4).

**Table 4:** Subgroup analysis of the association of TyG index with outcomes.

Subgroup	Neurological outcome OR (95% CI)	*p*-value	Functional outcome OR (95% CI)	*p*-value
Age				
<65 years	1.65 (1.02–2.67)	0.04	1.49 (0.91–2.44)	0.12
≥65 years	2.45 (1.55–3.87)	<0.01	2.21 (1.34–3.65)	0.002
Gender				
Male	2.05 (1.26–3.34)	0.004	1.76 (1.05–2.95)	0.03
Female	2.20 (1.19–3.99)	0.01	2.06 (1.15–3.68)	0.02
Hypertension status				
Hypertension	2.34 (1.42–3.86)	<0.01	2.11 (1.26–3.53)	0.005
No hypertension	1.89 (1.09–3.28)	0.07	1.68 (0.97–2.92)	0.07
Diabetes status				
Diabetes	2.71 (1.61–4.54)	<0.01	2.45 (1.42–4.22)	<0.01
No diabetes	1.83 (1.14–2.93)	0.07	1.52 (0.93–2.49)	0.10
Smoking status				
Current smoker	2.58 (1.47–4.51)	<0.01	2.23 (1.26–3.94)	0.006
Never/former smoker	1.95 (1.19–3.20)	0.07	1.71 (1.01–2.89)	0.05

## 4 Discussion

Our study findings underscore the significant association between the TyG index and short-term neurological and functional outcomes in patients with AIS following IVT. The TyG index, a marker of insulin resistance and metabolic dysregulation, has emerged as a potential predictor in the context of stroke prognosis (12, 13).

Previous studies have also explored the relationship between the TyG index and cardiovascular outcomes. A systematic review and meta-analysis confirmed the association of the TyG index with the risk of stroke, including its subtypes (14). Our results align with this evidence, indicating that a higher TyG index is linked to poorer outcomes in AIS patients post-IVT (15). While previous studies have explored the association between TyG index and stroke risk or mortality, our study is among the first to specifically evaluate its prognostic value in AIS patients treated with IVT. Unlike prior meta-analyses that focused on general stroke populations, we assessed short-term neurological and functional outcomes in a well-defined cohort of IVT-treated patients, providing clinically relevant insights into TyG’s role in post-thrombolysis prognosis.

The role of physical activity levels in stroke risk has been examined in a large US population study, which found that different types, frequencies, and intensities of physical activity were associated with reduced stroke incidence (16). This underscores the importance of lifestyle factors in stroke prevention and could potentially influence the TyG index, as physical activity is known to improve insulin sensitivity and metabolic health (17). Our results are consistent with previous research findings, indicating that a higher TyG index is associated with poorer outcomes in AIS patients following IVT (18). Also, the role of inflammation in stroke risk, often in conjunction with insulin resistance, cannot be overlooked. The C-reactive protein-triglyceride-glucose index (CTI), which combines measures of inflammation and insulin resistance, has been identified as a novel marker predicting stroke incidence in hypertensive populations (12). This is consistent with the subgroup results of our study, our findings contribute to this understanding by highlighting the TyG index’s role in stroke prognosis, independent of other risk factors.

Recent advances in stroke therapy have focused on intravenous thrombolysis as the mainstay of treatment for AIS (19). The recovery of neurological function and prognosis of patients after intravenous thrombolysis are the key to the treatment outcome. Our study adds to this knowledge by identifying the TyG index as a potential predictor of treatment response and outcomes in these patients.

The potential mechanisms linking the TyG index to stroke outcomes may involve insulin resistance and its associated metabolic derangements. Insulin resistance is known to increase inflammation, impair endothelial function and promote atherosclerosis, all of which are key in the pathogenesis of stroke (20). Furthermore, the TyG index has been proposed as a comprehensive statistical measure that incorporates fasting triglyceride and fasting glucose levels, reflecting the body’s metabolic health more broadly (21). In the context of AIS, the TyG index may serve as a marker of the metabolic environment that influences the response to IVT. Metabolic factors are increasingly recognized as important in stroke prognosis, influencing both neurological recovery and functional outcomes (22). Our findings contribute to this understanding by highlighting the TyG index’s role in stroke outcomes, independent of other risk factors.

The importance of modifiable risk factors in stroke, such as hypertension, diabetes, and physical activity levels, has been emphasized by the World Stroke Organization (23). Our findings suggest that the TyG index, as a marker of metabolic health, could be an additional modifiable risk factor to consider in stroke prevention and management strategies (24). A recent study demonstrated that statin pretreatment may improve neurological outcomes in AIS patients receiving IVT, potentially through pleiotropic effects on endothelial function and inflammation (25). This supports our emphasis on metabolic and inflammatory status-as reflected by the TyG index-as important prognostic factors in thrombolyzed patients. Additionally, a meta-analysis highlighted the critical role of collateral circulation in determining stroke severity and treatment response (26). While our study did not directly assess collateral flow, the association between TyG index and poor outcomes may partly reflect impaired cerebrovascular reserve in patients with metabolic dysfunction, a hypothesis worth exploring in future imaging-based studies.

It is important to acknowledge the limitations inherent in our study. Firstly, the observational nature of our research restricts our ability to infer causality between the TyG index and stroke outcomes. Secondly, while we controlled for several confounders, unmeasured variables such as dietary habits and physical activity levels, which are known to influence insulin resistance, may have impacted our results. Additionally, our study’s sample size and the specific demographic characteristics of our participants may limit the generalizability of our findings. Lastly, our focus on short-term outcomes post-IVT means the long-term predictive value of the TyG index remains to be determined. Future studies with larger cohorts, longer follow-up periods, and a broader range of confounding factors considered are needed to validate and expand upon our findings.

## 5 Conclusion

In conclusion, our study demonstrates that the TyG index is significantly associated with short-term neurological and functional outcomes in AIS patients following IVT. These findings contribute to the growing evidence supporting the role of metabolic factors in stroke prognosis and highlight the potential utility of the TyG index in clinical practice for risk stratification and outcome prediction.

## Data Availability

The original contributions presented in the study are included in the article/supplementary material, further inquiries can be directed to the corresponding author.
